# Locomotor and respiratory muscle abnormalities in HFrEF and HFpEF

**DOI:** 10.3389/fcvm.2023.1149065

**Published:** 2023-10-27

**Authors:** Norman Mangner, Ephraim B. Winzer, Axel Linke, Volker Adams

**Affiliations:** ^1^ Department of Internal Medicine and Cardiology, Heart Center Dresden, Technische Universität Dresden, Dresden, Germany; ^2^ Laboratory of Molecular and Experimental Cardiology, Heart Center Dresden, Technische Universität Dresden, Dresden, Germany; ^3^ Dresden Cardiovascular Research Institute and Core Laboratories GmbH, Dresden, Germany

**Keywords:** HFrEF, HFpEF, skeletal muscle, diaphragm, sarcopenia, cachexia

## Abstract

Heart failure (HF) is a chronic and progressive syndrome affecting worldwide billions of patients. Exercise intolerance and early fatigue are hallmarks of HF patients either with a reduced (HFrEF) or a preserved (HFpEF) ejection fraction. Alterations of the skeletal muscle contribute to exercise intolerance in HF. This review will provide a contemporary summary of the clinical and molecular alterations currently known to occur in the skeletal muscles of both HFrEF and HFpEF, and thereby differentiate the effects on locomotor and respiratory muscles, in particular the diaphragm. Moreover, current and future therapeutic options to address skeletal muscle weakness will be discussed focusing mainly on the effects of exercise training.

## Introduction

Heart failure (HF) is a chronic and progressive syndrome affecting worldwide billions of patients. Exercise intolerance and early fatigue are hallmarks of HF patients either with a reduced (HFrEF) or a preserved (HFpEF) ejection fraction. This is associated with a reduced quality of life. Furthermore, exercise intolerance, objectively measured as reduced peakVO2 during cardiopulmonary exercise testing, was identified as a prognostic marker for the risk of hospitalization for heart failure and death (1, 2) with a peakVO2 < 14 ml/kg/min leading to 5.6-fold increased risk of major cardiac adverse events (3). In contrast to initial suggestions, several studies performed in the 1980s and 1990s revealed that peakVO2 is poorly correlated to markers of central hemodynamics such as resting left ventricular ejection fraction (LV-EF) or even directly measured pulmonary wedge pressure and cardiac output at rest and during exercise (4–6). This indicates that other mechanisms than the central hemodynamics contribute to the impaired physical performance. In this review we discuss the role of HF-associated myopathy of both the locomotor and respiratory muscles contributing to exercise intolerance and reduced quality of life in HFrEF and HFpEF. We summarize five selected mechanisms for limb and diaphragm muscle loss and weakness in HF including (i) muscle mass, (ii) contractile dysfunction, (iii) fiber type composition and capillarization, (iv) mitochondria as well as (v) inflammation and reactive oxygen species. For this, we performed a literature search including the terms HFrEF and HFpEF combined with skeletal muscle, locomotor muscle, respiratory muscle and/or diaphragm with regard to clinical outcome and the before mentioned five selected molecular mechanisms. Relevant studies were identified by NM, EBW and VA, and references as well as citations of those articles were checked for further information.

## Influence of HF on locomotor muscles

**Figure 1:**
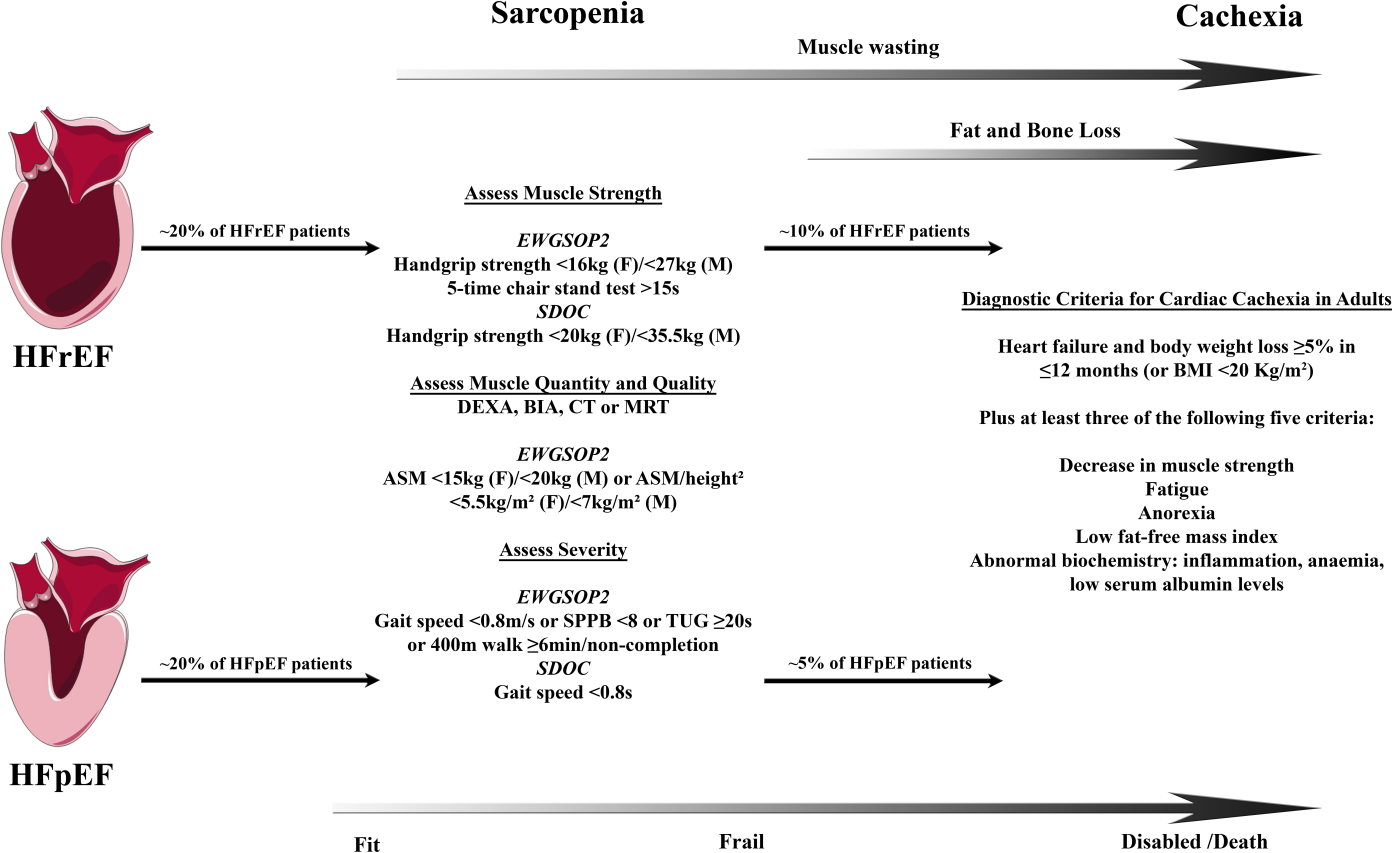
Wasting continuum in heart failure. Both HFrEF and HFpEF leads to loss of muscle quality and quantity followed by loss of fat and bone tissue in a substantial proportion of HF patients. Definitions of sarcopenia and cachexia may differ between countries and societies leading to some uncertainties in the prevalence of those conditions. Clinically, sarcopenia is associated with frailty leading into disability and increased mortality with cachexia. Prevalence for sarcopenia in HFrEF and HFpEF from Bekfani et al. (13), cachexia in HFrEF from von Haehling et al. (14), and cachexia in HFpEF from Pocock et al. (15). Definitions of sarcopenia according to the consensus definitions by the European Working Group on Sarcopenia in Older People 2 (EWGSOP2) in 2018 (16) and the Sarcopenia Definition and Outcomes Consortium (SDOC) in 2020 (17). Definition of cachexia according to Evans et al. (18). ASM indicates appendicular skeletal muscle; BIA, bioelectrical impedance analysis; CT, computed tomography; DEXA, dual-energy x-ray absorptiometry; F, female; HFrEF, heart failure with reduced ejection fraction; HFpEF, heart failure with preserved ejection fraction; M, male; MRI, magnetic resonance imaging; SPPB, short physical performance battery; and TUG, timed-up-and-go.

Whereas the myocardium and the muscular wall of the vasculature are responsible for organ perfusion and the respiratory muscles for the ventilation of the lungs, the locomotor skeletal muscles, the largest organ by mass in the human body, allow upright posture and body movement including nearly all activities of daily living. The lean mass of the limbs, e.g., both arms and legs, (appendicular lean mass) as determined by dual energy X-ray absorptiometry (DEXA) or other imaging methods approximates appendicular skeletal muscle mass and accounts for a large proportion of total skeletal muscle mass (7–9). With healthy ageing, a continuous decrease in total and appendicular muscle mass has been shown in different cohorts (8–12). In contrast, sarcopenia is now recognized as a muscle disease with the ICD-Code M62.5 and is in particular defined by a decline in muscle strength/quality but also quantity (Figure 1) (16, 17). The prevalence of sarcopenia increases with age, affects up to 50% of all octogenerians, and is associated with functional impairment and disability resulting in a loss of autonomy, quality of life, increased risk of falls and fractures, frailty, and premature death (19, 20). Muscle wasting, as a disease related process of accentuated muscle loss in discrimination to sarcopenia and physiological decrease in muscle mass in healthy ageing, can be exacerbated by an acute or chronic disease setting (21, 22). Having in mind that rapid skeletal muscle atrophy in healthy young men was already evident after two days of limb immobilization with a rate of 0.8% muscle loss per day over a period of one week (23), a recent meta-analysis of 35 trials compared the effect of muscle disuse on the time course of muscle atrophy of the lower limb in healthy volunteers, patients with ankle fracture, and critically ill patients, who were treated on an intensive care unit. The analysis revealed that disuse muscle atrophy occurred rapidly, with the highest rate of muscle loss in the most acute phase, and that these changes were least in healthy subjects and most severe in critically ill patients (24). This indicates that in acute as well as in chronic disease settings muscle dysfunction and wasting is not only a result of disuse and deconditioning rather the catabolic impact of illness, starvation, infection, and inflammation (25). Disuse atrophy in healthy subjects means a reduction only in muscle fiber size whereas muscle wasting/sarcopenia is also associated with a decrease in number of muscle fibers with different mechanisms involved in these processes (22). Among other chronic diseases, muscle wasting has been identified in cancer, chronic infection, chronic kidney disease, chronic obstructive pulmonary disease as well as HF (21). Muscle loss starts in early disease stages of HF and precedes the wasting process of fat tissue (8, 26). Once the overall wasting process goes along with a reduction in body weight this systemic metabolic disorder is termed cachexia, independent from the predominantly affected body compartment of muscle vs. fat tissue (27). This so-called wasting continuum in HF is depicted in Figure 1.

### Clinical data regarding locomotor muscle dysfunction in HF

#### HFrEF

Central hemodynamics can only partially explain the impaired exercise tolerance in HFrEF patients. Since the increase in oxygen uptake with physical exercise largely depends on the capability of skeletal muscle fibers to produce high-energy phosphates in an oxidative process, once again hemodynamics came into focus in an elegant study by Wilson and coworkers when the impact of reduced skeletal muscle perfusion was analyzed as rate limiting element of oxygen uptake and exercise tolerance (28). Cardiac output and leg blood flow was invasively measured in 34 HFrEF patients and 6 healthy controls during rest and physical exercise. HFrEF patients experienced marked exertional fatigue at peak exercise as well as significantly lower cardiac output and peakVO2 compared to healthy controls. However, one quarter of HFrEF patients developed exertional fatigue despite normal leg blood flow during exercise associated with lower blood oxygen extraction but comparable lactate production compared to patients with impaired leg blood flow indicating that intrinsic metabolic dysfunction of the skeletal muscle itself but not oxygen delivery *per se* limits oxygen uptake in these patients (28). In fact, several studies identified morphological and metabolic-functional alterations of skeletal muscles in HFrEF patients associated with impaired functional capacity such as reduced number of muscle fibers and their cross sectional area, changes in fiber type composition, increased fat infiltration and fibrosis, lower capillarization, impaired mitochondrial number and function, and altered bioenergetics, that will be discussed in detail later on (5, 26, 29–32). The presence of muscle wasting in HF patients, irrespective of underlying pathophysiological mechanisms, was found to be significantly associated with reduced peakVO2 independent of age, sex, NYHA class, LV-EF, or comorbidities in the SICA-HF study (33).

An important study published by Anker and colleagues in 1997 for the first time demonstrated in a prospective cohort of 171 patients with HFrEF that not only functional impairment as determined by reduced peakVO2 but also cachexia defined as documented non-oedematous and non-intentional weight loss of more than 7.5% of body weight over a period of at least 6 months is a strong and independent risk factor for mortality with a survival rate in cachectic patients of only 50% at 18 months. The risk of all-cause death was found to be 3-times higher in cachectic patients compared to those with stable weight or weight gain (34). However, different definitions of cachexia might lead to differences in the prevalence of the disease as well as to diverse power in outcome prognostication (18). This study did not distinguish if weight loss was caused by a reduction in muscle mass, fat tissue, skeletal mass, or a combination of them. In contrast, the longitudinal Health ABC study analyzed body composition trajectories in older adults with annually performed DEXA scans assessed serially over a period of 6 years. Patients who developed incident HF during follow-up had higher total body mass at baseline compared to those who did not. After developing HF, a disproportionate loss of total lean body mass and appendicular lean mass occurred, particularly among men. Fat mass, in contrast, remained stable in early disease state (8). Several studies performed in the last two decades consistently showed that mild to moderate obesity—even if associated with increased risk for cardiovascular diseases and all-cause mortality in healthy subjects—prevents early death in chronic HF. This phenomenon has also been shown for a plenty of other cardiovascular disease conditions and is termed the ‘obesity paradigm’ (35, 36). It has been clearly shown that muscle wasting/sarcopenia, which affects 20%–50% of patients with chronic HF including patients in young age groups, even before a reduction in total body weight sets in, relates with impaired exercise tolerance, quality of life and mortality (33, 37–39).

#### HFpEF

Even though obesity is highly prevalent in HFpEF and seems to be of causative impact at least in a subset of HFpEF patients with a distinct obesity phenotype, the so called obesity paradigm indicating higher mortality in patients with lower BMI has been shown consistently in different HFpEF populations (36, 40–42). Recently, this was confirmed in a secondary analysis of the DELIVER trial analyzing safety and efficacy of the SGLT2 inhibitor dapagliflozin vs. placebo in 6,263 patients with HFpEF according to baseline BMI. With BMI studied as a continuous variable the lowest risk of mortality was found in the BMI range between 25 and 35 kg/m² with a sharp increase in the group of patients with a BMI < 25 kg/m² (43). The prevalence of cachexia in HFpEF is not well established since patients with preserved LV-EF were underrepresented in most studies with broad LV-EF spectrum, and specific studies of large HFpEF cohorts are lacking (27, 34, 37). Therefore, a secondary analysis of the CHARM program which evaluated the impact of candesartan vs. placebo in a HFrEF, HFmrEF and HFpEF cohort is of value. Between baseline and 6 month-follow-up a loss of more than 5% body weight was found in 7%–8% of patients in all LV-EF subgroups. Weight loss was associated with excess mortality irrespective of baseline BMI or LV-EF (15). A study from Japan performed DEXA scans after discharge from HF hospitalization to analyze body composition. Nearly half of the patients had an LV-EF > 40%. Reduced appendicular skeletal muscle mass was found in 54% of the patients without any difference between HFrEF and HFpEF. Reduced muscle mass as well as lower fat mass was associated with worse prognosis in this apparently non-obese cohort (mean BMI 22 kg/m²) (39). The prevalence of muscle wasting in the HFpEF cohort of the SICA trial was lower with 19.7% (19.5% in patients with HFmrEF or HFrEF), which was clearly related to lower peakVO2, muscle strength and quality of life (13). The comparison of older HFpEF patients with age-matched healthy controls revealed that percent total lean mass and leg lean mass is significantly reduced in HFpEF patients and outreaches by far the extent of sarcopenia in normal aging. Again, the clinical relevance was demonstrated by a significant correlation of percent total lean mass and leg lean mass with peakVO2 and physical functional performance (44). That it is not only a matter of skeletal muscle mass but also of muscle function was indicated by a markedly reduced slope of the relationship of peakVO2 with percent leg lean mass (44). These studies underscore that muscle wasting is prevalent not only in HFrEF but also in HFpEF even though pathophysiological mechanisms in HFrEF and HFpEF partially differ (45).

It is still a matter of debate if weight loss—when intended and a result of a reduction in fat mass but not muscle mass—might improve cardiac function, exercise capacity and quality of life. Interestingly, abdominal obesity defined as an elevated waist circumference in patients with HFpEF from the TOPCAT trial was associated with higher all-cause mortality compared to those without abdominal obesity even when adjusted for BMI and despite the consistent finding of the protective effect of higher BMI on fatal events (obesity paradigm) (46). Further randomized clinical trials are warranted to determine the impact of intended weight loss, e.g., induced by caloric restriction or glucagone-like peptid 1 receptor agonists, and most susceptible patient subgroups since localization of fat tissue seems to be relevant (34, 41, 47, 48). Haykowsky and coworkers identified an altered skeletal muscle composition in older HFpEF patients with a loss of muscle fibers and replacement by intramuscular fat infiltration which was an independent predictor of peakVO2 indicating that muscle composition contributes to exercise intolerance in HFpEF (49).

Not LV-EF predicts muscle wasting, muscle function, and exercise capacity in HF, but functional parameters of the right ventricle (RV) have been shown to be predictive for both adverse outcome in chronic HF and exercise tolerance, cardiac function and body composition. Pravio et al. identified tricuspid annular plane systolic excursion (TAPSE) but not LV systolic function as an independent determinant of peakVO2 in a cohort of 362 HF patients irrespective of LV-EF below or above 40% (50). Right ventricular dysfunction was also related to the presence of weight loss in a stepwise association of the grade of RV dysfunction and the likelihood of cachexia with a relatively stronger link of cachexia to RV than LV dysfunction (27, 51). Determined by DEXA scan not lean mass but fat mass was reduced in patients with cachexia and significantly related to the degree of RV dysfunction (51). This finding raises the question if RV dysfunction also mediates skeletal muscle function and wasting or rather serves as a marker for advanced disease state and wasting of fat tissue. Experimental data in right ventricular HF indicate mitochondrial impairment of skeletal muscle (52) whereas improvement in RV function with homoarginine supplementation was shown in a HFpEF model in the absence of any impact on skeletal muscle force or cross-sectional fiber area (53). Future studies need to address the impact of RV function on skeletal muscle in HF and related therapeutic options.

## Influence of HF on respiratory muscles

The diaphragm is both among the largest muscles in humans and the main respiratory muscle responsible for normal ventilatory behaviors (54). Motor neural activation leads to diaphragmatic contraction causing pleural pressure to become more negative and abdominal pressure to be more positive (the difference is the transdiaphragmatic pressure, which increases during inspiration) (55). Relaxation of the diaphragm allows for elastic recoil of the chest wall, reducing thoracic cavity size, increasing intrathoracic pressure, and causing exhalation (55). However, in addition to inspiration, activation of the diaphragm muscle is necessary for expulsive behaviors, including expectoration and sternutation, which are essential for clearing the airways and maintaining airway patency (55). The diaphragm also plays an important role in speech, swallowing, and abdominal straining, and substantially supports blood return to the heart and lymph return to the thoracic duct from the abdominal lymph vessels during inhalation (56).

The clinical examination of the diaphragm can be done by imaging and functional tests. Imaging includes chest x-ray, fluoroscopy, and magnetic resonance tomography (57). Ultrasound is an inexpensive and readily available modality that provides data related to the diaphragmatic function, e.g., excursion, thickness, and thickening and can be used during exercise (56). Functional test can be divided into voluntary maneuvers, e.g., measurement of maximum static inspiratory pressure (PI_max_), and evoked maneuvers, e.g., magnetic or electrical phrenic nerve stimulation (58).

### Clinical data regarding respiratory muscle dysfunction in HF

#### HFrEF

Initial descriptions of inspiratory muscle involvement in HFrEF dates back to the 1990s with a small study showing that patients with HFrEF had lower Pi_max_ compared with healthy controls (59). A strong correlation was found for ratings of perceived dyspnea and PI_max_ values underlining the potential clinical importance of diaphragm dysfunction in HFrEF. Using invasive measurement of the transdiaphragmatic pressure following electrical or magnetic phrenic nerve stimulation, it was shown that patients with HFrEF exhibit impaired contractility of the diaphragm as compared to healthy individuals (60). Recently, a study of end-stage HFrEF patients documented a significant reduction of Pi_max_ and expiratory pressure (Pe_max_) by 38% and 25% in HFrEF compared with patients undergoing coronary artery bypass surgery without signs of HF (61). Notably, diaphragm thickness, which was measured by ultrasound showing a 23% lower mean value in HFrEF, was significantly correlated with Pi_max_ in this study (61). Diaphragm ultrasound has occurred as a novel tool for assessment of diaphragm function (62) and has been shown to be impaired and associated with reduced exercise capacity in HFrEF patients (63).

With regard to morbidity and mortality, different studies have shown that diaphragm weakness is common in patients with HFrEF (64), its degree of reduction is correlated with worsening NYHA functional class (65), and is an independent predictor of mortality (66, 67). Notably, Pi_max_ also adds prognostic value beyond known risk factors of clinical deterioration, including peakVO2, LV-EF, and norepinephrine plasma concentration (66).

#### HFpEF

Clinical data on respiratory muscle affection in HFpEF patients are still limited. Spiesshoefer et al. examined 22 HFrEF and 8 HFpEF patients and compared them to 19 healthy matched controls showing significantly lower forced vital capacity, Pi_max_, Pe_max_ and diaphragm strength (twitch transdiaphragmatic pressure in response to supramaximal cervical magnetic phrenic nerve stimulation) in both HFrEF and HFpEF compared with controls (68). In a Japanese patient cohort including 1,023 HFpatients (445 HFrEF, 578 HFpEF), respiratory muscle weakness was defined as Pi_max_ <70% of predicted value. Applying this cut-off, 190 (42.7%) HFrEF and 226 (39.1%) HFpEF patients had respiratory muscle weakness, which was independently associated with all-cause mortality in both HFrEF (adjusted HR 2.13, 95%-CI 1.17–3.88) and HFpEF (adjusted HR 2.85, 95%-CI 1.74–4.67) (69). A recent meta-analysis on the effect of inspiratory muscle training in HFpEF revealed a substantial improvement of peak oxygen consumption and 6-min walk distance indicating that inspiratory muscle weakness is present in HFpEF and contributes to a reduced cardiorespiratory fitness (70).

## Molecular data regarding locomotor and respiratory muscle dysfunction in HF

**Figure 2:**
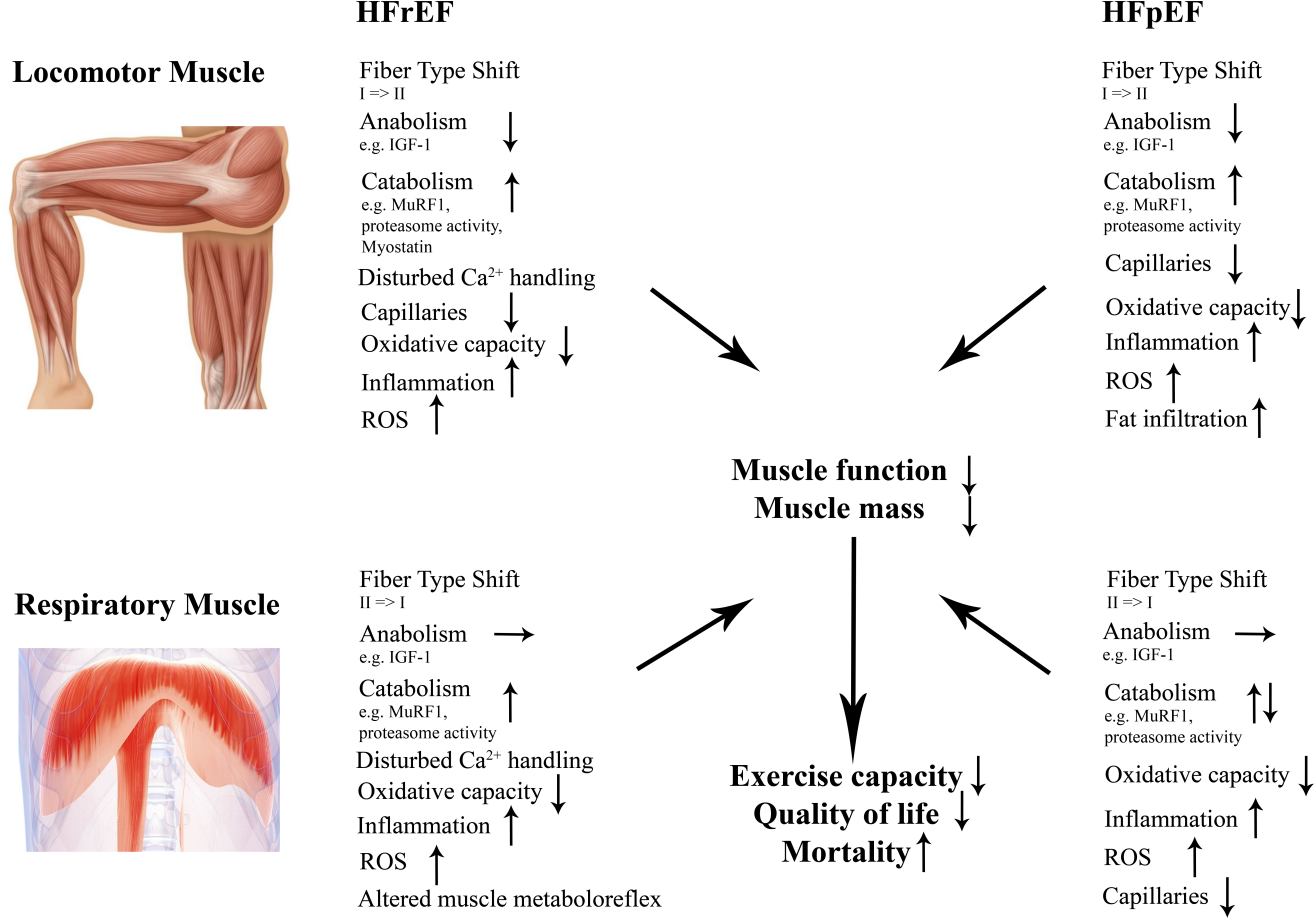
Structural and molecular changes in locomotor and respiratory muscles in HFrEF and HFpEF. Those changes contribute to impaired muscle mass and function in both entities contributing to a worse outcome. HFrEF indicates heart failure with reduced ejection fraction; HFpEF, heart failure with preserved ejection fraction; IGF-1, insulin-like growth factor 1; MuRF1, muscle ring finger protein 1; ROS, reactive oxygen species.

In the following chapters we will discuss molecular effects of both HFrEF and HFpEF on locomotor and respiratory muscles with regard to (i) muscle mass, (ii) contractile dysfunction, (iii) fiber type composition and capillarization, (iv) mitochondria as well as (v) inflammation and reactive oxygen species (Figure 2).

### Muscle mass

Loss of muscle mass is closely associated with functional impairment and reduced quality of life. Low skeletal mass independently predicts mortality in patients with chronic HF leading to a 4.5 times higher risk of 1-year all-cause mortality (71). Several factors may play important roles in triggering muscle wasting and atrophy in patients with HF. Proinflammatory cytokines like tumor necrosis factor alpha (TNF-α), interleukin-1 (IL)-1 and IL-6 are known to trigger muscle wasting/dysfunction and their expression is even normalized with anti-atrophic interventions like exercise training (72, 73). Nevertheless, muscle atrophy as such is the result of an imbalance between protein synthesis and degradation. One of the most important system in the skeletal muscle for protein degradation is the ubiquitin-proteasome system (UPS). This enzymatic process involves the binding of ubiquitin to the target protein before it is degraded by the 26S proteasome (74–76). The rate limiting and protein specific step in this process are the E3 ubiquitin protein ligases (14). The most investigated and characterized E3 ubiquitin-ligases in the skeletal muscle are MuRF1 (also known as Trim 63) and MafBx (also known as atrogin-1 or FBox-32). They were first described by Dr. Bodine performing transcript profiling to screen for candidate molecular mediators of muscle atrophy (77). The importance of MuRF1 and MafBx for induction of muscle atrophy was further supported by showing that induction of muscle atrophy was attenuated in mice deficient in either MuRF1 or MafBx (77, 78). Besides the UPS, myostatin (GDF-8) is another important regulator of muscle mass. Myostatin, a negative feedback regulator of muscle growth, is generated by skeletal myofibers, circulates in the blood and signals back to the myofibers to suppress muscle growth (79). This regulatory function of myostatin on muscle mass is directly supported by animals lacking the myostatin gene (80, 81), or animals treated with the myostatin inhibitor follistatin (82) or YK11 (83). Also in humans, mutations in the myostatin gene resulted in increased muscle mass and force development (84). Besides the activation of catabolic processes, also the inhibitions of anabolic pathways are resulting in muscle atrophy, which is sometimes referred as anabolic resistance (85). One of the most potent anabolic factor is insulin-like growth factor 1 (IGF-1). IGF-1 increases skeletal muscle protein synthesis via PI3K/Akt/GSK3ß pathways and inhibits the FoxO mediated activation of E3 ubibiquitin ligases (86). This powerful action of IGF-1 is evident in IGF-1 transgenic animals where the local expression in the skeletal muscle resulted in muscle hypertrophy associated with elevated force generation (87, 88).

#### HFrEF

HFrEF patients often present with muscle atrophy. In the before mentioned SICA-HF trial (33) including 200 HFrEF patients, the prevalence of muscle wasting (quantified by DEXA) was around 20% compared to healthy controls. A meta-analysis including 11 different studies with 1,742 patients documented that the pooled prevalence for sarcopenia in patients with HF was 34% ranging from 10% to 69% (89). It was even evident that patients with muscle wasting showed a greater impairment in LV-EF and a reduced exercise capacity. Furthermore, skeletal muscle mass is an independent predictor of mortality in HFrEF patients and a recently published study also concluded that the percentage of intramuscular fat may have adverse consequences in HFrEF patients (90). The association between the development of HFrEF and muscle atrophy was also documented in animal models of HFrEF (91–94). With respect to the basic underlying mechanisms, molecular analyses have been performed in human muscle biopsies (31, 95, 96) and skeletal muscle tissue obtained from animal models (94, 97).

One of the first study looking into molecular marker for muscle atrophy in human skeletal muscle biopsies was the study by Hambrecht and colleagues (96). They were the first documenting a significant reduced expression of IGF1 in skeletal muscle biopsies obtained from HFrEF patients compared to healthy age matched controls. Furthermore, a close correlation between local IGF1 expression and muscle cross-sectional area, a sign for muscle atrophy, was documented. An additional study analyzing human skeletal muscle biopsies from the *M. vastus lateralis* with respect to the expression of anabolic and catabolic protein expression was the LEICA study performed by Gielen and colleagues (31). A significant upregulation of MuRF1 protein expression was detected in biopsies from HFrEF patients compared to healthy controls with no changes in MafBx expression. In addition, a significant reduced expression of IGF1 was observed. Nevertheless, the role of the UPS system for skeletal muscle atrophy in humans with HFrEF remains inconclusive with a study reporting no altered expression of UPS components in HFrEF muscle biopsies when compared to healthy controls (98).

Looking into animal models of HFrEF [genetic models, coronary artery ligation, monocrotalin injection, transverse aortic constriction (TAC) operation] provides a more detailed and clearer conclusion. Several studies in the current literature documented an upregulation of MuRF1, MafBx or the proteasome activity in the skeletal muscle of animals presenting with chronic HF (92, 94, 97, 99, 100). The relevance of MuRF1 for the induction of muscle atrophy in HFrEF is further supported by specific inhibition studies (101). Treating HF animals with a small molecule targeting the central coil domain of MuRF1 inhibited the development of muscle atrophy and dysfunction.

Regarding the expression of myostatin in the locomotor skeletal muscle of HFrEF only a few reports are available. Increased expression of myostatin was detected in animal models (102, 103) or muscle samples of HFrEF patients (104). The muscular expression may be regulated by the increased level of TNF-α and p38MAK-dependent pathways (102) and incubating skeletal muscles cells with myostatin exhibited an increase in proteolysis and an increased expression of MuRF1 and MafBx (105).

With regard to respiratory muscles and compared to controls, HFrEF patients presented with diaphragm wasting as indicated by a reduced diaphragm thickness measured by ultrasound and severe fiber atrophy in histologically examined specimens (61). As described for the locomotor muscles, the UPS is the major proteolytic pathway responsible for protein degradation in pathological muscle loss (106). Thus, a 46% higher muscle-specific E3 ligase MuRF1 protein expression was evident in the diaphragm of HFrEF patients compared to controls accompanied by a 54% higher protein ubiquitination at lysine 48 residues, with proteasome activity elevated by 54%. MuRF1 protein expression was correlated with clinical parameters (diaphragm thickness (*R* = −0.46, *p* = 0.015), peakVO2 (*R* = −0.43, *p* = 0.024), VE/VCO2 (*R* = 0.40, *p* = 0.045)) in this study implicating that diaphragm weakness and atrophy of the diaphragm in HFrEF is underpinned by MuRF1-dependent ubiquitin-proteasome degradation (61). In an animal model of HFrEF, those findings could be replicated and fiber atrophy as well as diaphragm weakness were obvious (107). Treatment with a small molecule potentially targeting MuRF1/MuRF2 was able to attenuate muscle wasting and to preserve diaphragm function (107). Regarding anabolic factors, no difference between IGF-1 mRNA expression was found in the diaphragm of HFrEF animals compared with controls (97).

Those impairments of diaphragm muscle mass and function are remarkable since limb muscle weakness in HFrEF might also be triggered by disuse due to a reduced exercise capacity and the lack of physical activity. In contrast, the diaphragm in HF is even encountered by an increased workload due to dyspnea (61, 65) and, therefore, experiences molecular and structural effects comparable to those seen after aerobic exercise training, e.g., increased activity of antioxidant and metabolic enzymes (100) or, as discussed below, a fiber type shift towards slow-twitch type 1 fibers (61, 108). Thus, the diaphragm might serve as a model which is independent of disuse to elicit the mechanisms of HF associated myopathy.

#### HFpEF

Detailed muscle analyses revealed that in HFpEF patients myofibers were replaced by fat incorporation with a 30% increase in fat content in the skeletal muscle of HFpEF patients (49). This increase in myosteatosis in HFpEF was also seen in the study by Weiss et al. (109) showing a 2 or 4-fold higher fat content in the skeletal muscle when compared to HFrEF and healthy controls, respectively. Also in an established animal model of HFpEF (Zucker fatty/spontaneously hypertensive heart failure F1 hybrid rat (ZSF1)), the increased incorporation of fat into the skeletal muscle was documented (110). This increase in myosteatosis in HFpEF may impact on exercise performance through several mechanism's like reducing oxygen delivery to the active muscle, impairing mitochondrial function or having catabolic effects via cytokines secreted by adipocytes (111).

In contrast to morphological changes occurring in the skeletal muscle of HFpEF, molecular alterations contributing to muscle atrophy are less investigated. One reason for this discrepancy may be the availability of an animal model fully reflecting the human situation. Comparing at least 3 different animal models with the human situation, Goto and colleagues concluded that none of the investigated HFpEF animal model is 100% showing the muscular changes as observed in humans, but the ZSF1 obese rat is the model that come closest to the human situation (112).

With respect to the analysis of molecular pathways regulating muscle mass in HFpEF so far two human (95, 113) and several experimental studies using either the ZSF1 obese or Dahl Salt-sensitive (DSS) rat model are available (97, 114). Summarizing the results from all the human and animal studies, we see an upregulation of the ubiquitin E3-ligases Murf1 and MafBx, an elevation in protein ubiquitination and an increase in autophagy in the HFpEF skeletal muscle tissue when compared to healthy controls. Assessment of proteasome activity revealed inconclusive results. In human skeletal muscle tissue an elevated activity was evident (95), whereas in the DSS rat model no difference was noted between HFpEF and controls (97, 114).

Myostatin, another regulator of muscle mass, was only investigated in the study by Bekfani and colleagues. They reported an increased mRNA level in the HFpEF patients when compared to healthy controls (113). With respect to the anabolic factor IGF-1 a significant lower expression was noted in human skeletal muscle biopsies obtained from HFpEF patients (113).

Data on respiratory muscle changes in HFpEF are still scarce and inconclusive. In the HFpEF animal model of DSS rats, which is a hypertension induced model of HFpEF, a reduced cross-sectional area of both type 1 and type 2 fibers of the diaphragm was detected indicating muscle atrophy. This was accompanied by a reduced specific force development and a higher fatigability of the diaphragm fiber bundles in HFpEF animals (114). In contrast, in the HFpEF animal model of obese ZSF1 rats, the cross-sectional area of type I and type IIa fibers increased, whereas the one of type IIb/IIx fibers decreased, which led to preserved muscle mechanics except a higher relative fatigue in HFpEF animals (115). Those differences between models can be explained by the co-morbidity of obesity and its associated chronic respiratory loading, which can act as a training stimulus to increase fiber size (116, 117), Those disparities also raises the question, which animal model comes closest to the clinical syndrome of HFpEF in humans. With regard to locomotor skeletal muscle, a recent, above mentioned study aimed to investigate molecular changes of 3 different animal models [DSS rat, ZSF-1 rat, and the TAC/deoxycorticosterone acetate (TAC/DOCA) mouse] and to compare them with alterations occurring in muscle biopsies obtained from human HFpEF patients (112). It was found that protein expression between skeletal muscle tissue obtained from HFpEF patients and the ZSF1 rats revealed similarities for protein markers involved in muscle atrophy (MuRF1 expression, protein ubiquitinylation, and LC3) and mitochondrial metabolism (succinate dehydrogenase and malate dehydrogenase activity, porin expression). The other two animal models exhibited far less similarities to the human samples suggesting ZSF1 as a suitable model to study pathophysiological and treatment effects in HFpEF (112).

Regarding anabolic and catabolic factors in the diaphragm of HFpEF animals, no difference was found for IGF-1 mRNA expression compared to controls (97), whereas the expression of catabolic factors is inconclusive with those studies finding an increased MuRF1 (and MuRF2) mRNA-expression (97) and those showing reduced MuRF1/2 expression as well as reduced proteasome activity (114, 118).

### Contractile dysfunction and calcium handling

Muscle fatigue and weakness in HF are not only the result of an imbalance of protein synthesis and degradation, but can also be caused by contractile dysfunction due to oxidative modifications of key contractile proteins and as the consequence of impaired excitation-contraction coupling (85, 119). Those changes are typically reflected as a reduction of specific force development, e.g., absolute force normalized to muscle cross-sectional area, whereas atrophy-related muscle weakness is characterized by an absolute force reduction.

Calcium homeostasis is guaranteed by a tight control between calcium release and uptake. The main channel for calcium release in the skeletal and cardiac muscle from the sarcoplasmatic reticulum (SR) is the ryanodine receptor 1 (RYR1) complex, whereas the family of the sarco-endoplasmic reticulum Ca^2+^ ATPase (SERCA) is responsible for the re-uptake (120, 121).

#### HFrEF

In experimental HF, cytosolic Ca^2+^ shifts are reduced in both slow- and fast-twitch locomotor muscles (122, 123). For example, the extensor digitorum longus (which is a typical fast-twitch skeletal muscle) showed prolonged Ca^2+^ transients and reduced SR release in HF rats, which was associated with reduced muscle performance and accelerated fatigue development (124). In both animal models (125) and human muscle biopsies (126), a reduced expression of SERCA proteins is evident in locomotor muscles, which indicates reduced Ca^2+^ sequestration into the SR. With regard to Ca^2+^ release, dysfunction of the RYR1 complex is thought to be a main contributor for a disturbed homeostasis in HF (120, 123, 127, 128). For example, binding of FKBP12 (also known as calstabin) to RYR1 is reduced in HF leading to a leaky RYR1 complex with increased Ca^2+^ efflux from the SR (120). This might be caused by hyperphosporylation of RYR1 by protein kinase A under the circumstances of chronic β-adrenergic signaling in experimental HF (127) which is also evident in *M. vastus lateralis* biopsies of HF patients (129). Overall, Ca^2+^ overload might lead to further detrimental consequences including contractile dysfunction due to a reduced Ca^2+^ fiber sensitivity (130), mitochondrial dysfunction due to mitochondria Ca^2+^ overload (since mitochondria act as a buffer) (131) and accelerated atrophy via activation of protein degradation systems including calpains (132) and consequently the UPS (97, 119). Moreover, higher cytosolic Ca^2+^ concentration leads to increased ROS production from the key sources in skeletal muscles including mitochondria, NADPH oxidase (Nox), and xanthine oxidase (100). For example, inhibition of xanthine oxidase in mice with HF prevented the atrophy of type I and type II fibers in locomotor muscles and preserved exercise capacity (133).

A disturbed Ca^2+^ homeostasis is also evident in the diaphragm in the setting of HF (134). Comparable to the locomotor muscles, a reduced FKBP12 binding to the RYR1 was described in the diaphragm of HF patients (61). In addition, sensitivity of single diaphragm muscle fibers to cytoplasmic Ca^2+^ concentrations is decreased in HF patients (61, 130). In contrast to the locomotor muscles, expression of SERCA1 and SERCA2a were higher in diaphragm biopsies from HF patients, indicating a divergent response between limb and respiratory muscle (61).

Upregulation of Nox, in particular Nox 2 and Nox 4, has been described for diaphragm biopsies of HF patients (61, 135), accompanied by greater protein oxidation despite increases in antioxidant enzymes (135). The consequent increase in ROS production can lead to activation of catabolic factors, such as E3-ligases, resulting in diaphragm atrophy and post-translational oxidative modifications of sarcomeric proteins, which contribute to impaired diaphragm function (119). Targeting these sources of ROS might be of interest in the treatment of exercise intolerance in HF. For example, reduction in mitochondrial ROS production by application of a mitochondrial-targeted antioxidant (136) or a neutral sphingomyelinase inhibitor (137) preserved diaphragm dysfunction in HF rats post MI. Interestingly, certain isoforms of Nox seem to play different roles in diaphragm abnormalities during development of HF with knock out of Nox2 restoring diaphragm function early and late after MI (138, 139) whereas Nox4 knock out had no impact in on early diaphragm changes after acute MI (140) but restored function later on (139).

#### HFpEF

While there is pathophysiological evidence for a disturbed Ca^2+^ homeostasis in the myocardium of certain HFpEF etiologies (141), data on the role of Ca^2+^ homeostasis in HFpEF related skeletal muscle dysfunction are not examined and further studies are necessary.

#### Fiber type and capillarization

Skeletal muscle can be divided into two broad types based on fiber type—type I and type II muscle fibers. They differ with respect to metabolic and contractile attributes. Type I, also known as slow twitch or “red” muscle, is dense with capillaries, rich in mitochondria, and fatigue-resistant. Type II, or fast twitch muscle, has three major subtypes (IIa, IIx, and IIb) that vary in both contractile speed and force generation, but are generally more fatigable compared to slow twitch fibers. Therefore, changes in fiber type composition of the respective muscle will have important consequences for force generation and fatigability. The vascular bed in skeletal muscle functions mainly to supply oxygen to muscle fibers and capillary density strongly correlates with human skeletal muscle oxidative capacity (142). Therefore, it is also possible that the capillary density will be important in determining exercise tolerance in HF independent of other biochemical or histological changes in the skeletal muscle.

#### HFrEF

Since the first description of fiber type changes in the skeletal muscle of patients with chronic HF by Donna Mancini in the year 1989 (32), several other groups confirmed these results (30). In that pioneering work the authors analyzed skeletal muscle biopsies from 22 patients with HFrEF (peakVO2 15.4 ± 4.7 ml/kg/min; LV-EF 20 ± 7%) and eight normal subjects. Compared with normal subjects, patients with HFrEF exhibited a shift in fiber type distribution with increased rates of the fast twitch, glycolytic type IIb fibers; however, at the single fiber level, atrophy of those type IIa and type IIb fibers was evident (32). Importantly, the peakVO2 in HFrEF patients correlated with the percentage of type I fibers, whereas the correlation with type II A fibers was inverse (143). Therefore, a switch from slow to fast twitch fibers is now an accepted feature in the skeletal muscle of HFrEF patients. With respect to HFrEF animal models, the results are not so clear since some reported a reduction of type I fibers (144–146), whereas others not (147). Besides the change in fiber type composition, a reduction in the capillary–to-fiber ratio is also reported in the skeletal muscle of HFrEF. In an early work, Sullivan and colleagues investigated the *M. vastus lateralis* of 11 patients with long-term HF (LV-EF 21 ± 8%) and nine normal subjects (148). Using histochemical staining, they reported that in HFrEF patients the number of capillaries per fiber were decreased in type I and type IIa fibers. This observation was confirmed by several other groups (149), and also experimental models of HFrEF (like LAD ligation model or TAC) showed a decrease in skeletal muscle capillary density and capillary-to-muscle fiber ratio in HFrEF animals (150, 151).

In contrast to locomotor skeletal muscles the diaphragm behaves *vice versa* in HFrEF with regard to fiber type distribution. There are consistent data applying different molecular and histological techniques in animal models and HF patients showing a shift from fast twitch type II to slow twitch type I fibers (61, 108, 152) with the latter one accounting for 67% (95%-CI 57%–76%) in humans with HFrEF compared to 54% (95%-CI 44%–64%) in controls (61). This finding mimics results of exercise training in peripheral muscles of HFrEF patients (153) and might be caused by increased workload due to dyspnea (61, 65). This exercise-like effect is supported by increased activity of antioxidant enzymes and preserved markers of oxidative metabolism (100) as well as increased expression of other markers sensitive to an exercise stimulus (154), e.g., PGC1ɑ in the diaphragm of animal models and humans with HFrEF (61). In animal models of HFrEF, the increase in diaphragmatic blood flow during submaximal exercise was greatest in those animals with most severe HF (155). Presumably the increased blood flow was required for increased work of breathing. Contrary, a relevant deoxygenation appears to happen during exercise in accessory respiratory muscles measured by near-infrared spectroscopy (156). The reasons for that finding are incompletely understood and may include hypoperfusion due to a reduced cardiac output, changes in capillary-fiber ratio, and altered muscle metaboreflex. In particular, the latter one is an important mechanism since muscle metaboreflex is a key regulator of the cardiovascular exercise response and is triggered by metabolic byproducts (e.g., lactic acid) in skeletal muscles that stimulate afferent nerve fibers leading to sympathetic stimulation with a resultant increase in cardiac output and vasoconstriction of non-active muscles (157, 158). In a healthy subject, baroreceptor unloading during muscle metaboreflex activation results in an increase in mean arterial pressure primarily due to an increase in cardiac output, whereas in HF baroreceptor unloading during muscle metaboreflex activation increases mean arterial pressure primarily via vasoconstriction (56). In a canine model of HFrEF, baroreceptor unloading was simulated by bilateral carotid occlusion; this resulted in a pressor response caused by peripheral vasoconstriction of all vascular beds (including the ischemic active skeletal muscle) with no preferential vasoconstriction of the nonischemic vasculature suggesting that restoration of blood flow to ischemic active muscles is remarkably attenuated in HF because of the absence of preferential vasoconstriction of the nonischemic vasculature (159). Because the diaphragm is metabolically more active during exercise, altered diaphragm metaboreflex can contribute to limited exercise capacity in patients with HF.

#### HFpEF

Kitzman and colleagues were the first to report that also in skeletal muscle of HFpEF patients a fiber type shift occurs and that the capillary-fiber ratio is reduced (160). They examined *M. vastus lateralis* biopsies from 22 older HFpEF patients and compared them to 43 age-matched healthy controls. Histological analysis revealed that the percentage of type I fibers, type I-to-type II fiber ratio, and capillary-to-fiber ratio were reduced, whereas the percentage of type II fibers was higher in HFpEF. This reduction of type I fibers in the locomotor skeletal muscle of HFpEF patients was recently confirmed by Zamani and coworkers (161). Analyzing the fiber type distribution and capillary-to-fiber ratio in animal models of HFpEF, the results are not uniform. Depending on the animal model investigated different results were reported. Using the DSS rat model for HFpEF, Bowen et al. was the first to report no change in fiber-type composition in the soleus muscle when compared to controls (114). Switching the animal model to the ZSF1 obese model, Bowen and colleagues (162) as well as others (110, 115) could detect a fiber shift from I to IIa, and a lower capillary-fiber ratio.

Similar to HFrEF, patients with HFpEF are characterized by greater increases in leg muscle vascular resistance index and greater decreases in leg muscle blood flow index compared with controls during inspiratory resistive breathing (to activate the metaboreflex). Furthermore, respiratory muscle blood flow index responses normalized to pressure generation during inspiratory resistive breathing were exaggerated in HFpEF compared with controls (163). Those clinical findings have been proven in an obese-HFpEF rat model demonstrating blunted skeletal muscle blood flow during contractions in parallel to microvascular structural remodeling, fiber atrophy, and isotonic contractile dysfunction in the locomotor muscles (115). In contrast and as mentioned before, diaphragm alterations included type IIx fiber atrophy despite type I/IIa fiber hypertrophy, with increased indices of capillarity in the diaphragm (115). Alongside those fiber type specific changes, a general shift towards a more oxidative phenotype is evident in the diaphragm of HFpEF animals (118).

### Mitochondria

Mitochondria are the powerhouse of the cell and therefore alterations in their number and function will have significant impact on skeletal muscle function. Increasing evidence has shown that mitochondrial abnormalities, including altered metabolic substrate utilization, impaired mitochondrial oxidative phosphorylation, increased reactive oxygen species (ROS) formation, and aberrant mitochondrial dynamics, are closely related to HF. In recent years' new mitochondria-targeted treatment focused on enhancing mitochondrial function to improve heart and skeletal muscle contractility in patients with HF, making it fundamentally more effective at addressing energy problems (164, 165). Recently, the treatment of HFrEF patients with intravenous iron isomaltoside (FERRIC-HF II) showed a significant improvement in phosphocreatine (PCr) recovery time, an indirect measure for improved mitochondrial oxidative phosphorylation (166). Most studies analyzing muscular mitochondrial function in HF are performed in muscle biopsies from HFrEF patients and animal models of HFrEF. Far less information is available with respect to HFpEF.

#### HFrEF

Already in 1985, Wilson and colleagues concluded from gated phosphorus-31 nuclear magnetic resonance measurements for the first time that exertional fatigue in patients with HF may result in a greater than normal PCr depletion and/or acidosis in the working muscle (167). This observation was the first showing indirectly that mitochondrial energy metabolism is disturbed in the skeletal muscle of HFrEF patients. This assumption was confirmed by many authors analyzing the prevalence and morphometric characteristics of mitochondria as well as mitochondrial enzyme activities and mitochondrial oxidative phosphorylation in skeletal muscle tissue obtained from HFrEF patients or adequate animal models. Histological evaluations revealed numerous quantitative and structural alteration ranging from reduced number to reduced surface density and mitochondrial cristae (29, 148). The relevance of these morphological alterations is strengthened by the correlation between the volume density and peak exercise (29). In line with the morphological alterations in HFrEF, many studies reported reduced activity or expression levels of key enzymes of oxidative phosphorylation like citrate synthase (CS), succinate dehydrogenase (SDH), mitochondrial creatine kinase (mi-CK) and members of the respiratory chain (29, 148, 168, 169). Assessment of oxidative phosphorylation or mitochondrial chain respiratory capacity revealed some conflicting results. Studying a rat model of chronic HF (induced by aortic banding), muscles from HF rats exhibited a dramatic decrease in oxidative capacities of 35% for red soleus muscle and 45% for white gastrocnemicus muscle (150). This goes along with a reduction in mitochondrial enzyme activities with a parallel decrease in the mRNA level of cytochrome-c-oxidase (COX) I and IV, but no change in mitochondrial DNA content (170). Harvesting skeletal muscle tissue from HFrEF patients and from healthy controls and dividing the healthy controls into sedentary or active individuals revealed that muscle oxidative capacity was identical between HFrEF and sedentary controls and was different between HFrEF and active controls (171). These finding clearly stimulate the discussion if intrinsic skeletal muscle alterations in chronic HF patients are the result of a disease-specific myopathy or a result of deconditioning (172).

What are possible mechanisms leading to mitochondrial alterations in HFrEF? It was speculated for a long time that oxidative stress plays a pathological role in the development of HFrEF (173). Generating mice deficient for manganese superoxide dismutase exhibiting excess formation of oxygen radicals in the skeletal muscle tissue resulted in morphological changes of mitochondria and lower ATP production (174). In addition, myocardial infarction-induced HF leads to low mitochondrial oxidative enzyme activities, associated with pro-inflammatory transcription factors activation and low insulin signaling (175) that might directly impair skeletal muscle performance (176). Mitochondrial changes could also contribute to enhanced reactive oxygen species generation (177), which might *per se* further enhance inflammation, insulin resistance, further mitochondrial dysfunction, excitation-contraction uncoupling and ultimately an imbalance in muscle catabolism/anabolism.

Regarding respiratory muscles, early studies revealed swollen and degenerated mitochondria in the diaphragm of HFrEF rats (178), which was accompanied by impaired mitochondrial function (179). More specifically, a lower complex-I-activity with unaltered complex-II and—IV activity was detected in the diaphragm of experimental HFrEF animals (99). In humans with HFrEF, a higher expression of porin indicated a higher mitochondria content, which is in line with a shift towards an oxidative fiber type (61). However, a reduced state 3 and state 4 respiration across different substrates for complex I to IV in isolated mitochondria was evident in the diaphragm of HFrEF patients (61). Mitochondrial function was inversely correlated with ventilatory efficiency as measured by VE/VCO_2_ slope and positively correlated with exercise capacity measured by peakVO2 (61). Moreover, autophagy/mitophagy was assessed by evaluating the protein expression of LC3-I/LC3-II and p62. The ratio LC3-I/LC3-II was 1.4-fold higher in HF (*p* = 0.009), and p62 protein expression was 2-fold higher (*p* = 0.0014) in HFrEF compared with controls, a combination indicating inhibited autophagy/mitophagy (180).

Overall, these human data revealed that despite increased measures of overall number and content, intrinsic dysfunction of mitochondria is present in the diaphragm of HFrEF patients, potentially caused by impaired mitophagy (61).

#### HFpEF

So far only a few studies investigated mitochondrial metabolism and oxidative respiratory capacity in the skeletal muscle of HFpEF. The first hint that also in the skeletal muscle of HFpEF mitochondrial oxidative phosphorylation is impaired came from ^31^P-magnetic resonance spectroscopy (^31^P-MRS) studies. In the first study Bhella and coworkers used ^31^P-MRS on 2 HFpEF and 2 healthy controls to study mitochondrial energy metabolism (181). They clearly documented that oxidative phosphorylation ATP production rates were lower in HFpEF patients, anerobic glycolysis ATP production rates were greater in HFpEF patients, as was the PCr recovery time constant when compared with healthy controls. These results were confirmed by Weiss and colleagues in a larger cohort (109). While the finding from ^31^P-MRS studies were important in showing alteration in energy metabolism at the mitochondrial level, they did not directly assess mitochondrial content or oxidative capacity. The first study measuring mitochondrial function directly was performed by Bowen and colleagues (114). Using the DSS rat model, the diaphragm muscle showed impaired *in situ* mitochondrial respiration, indicating electron transport chain dysfunction. This impairment of mitochondrial oxidative phosphorylation was recently confirmed in the ZSF1 rat model of HFpEF (110, 182). The study of Kelley and colleagues demonstrated that in locomotor muscles, HFpEF development decreased specific force by 30% and maximal mitochondrial respiration of complex I and II by 40%–55%. In addition, a tendency towards an increase in mitochondrial oxygen radical formation was noted, nourishing the speculation that also in HFpEF oxygen radicals are central trigger for muscular dysfunction (182). Also in the ZSF1 obese rat model a significant lower oxygen consumption, mainly in mitochondrial complex-IV, was seen in the ZSF1-obese animals when compared to the ZSF1-lean counterparts (110).

The alterations in mitochondrial oxidative capacity are supported by molecular alterations detected in human muscle biopsies (112, 183) or muscle samples obtained from experimental models (110, 114, 184). Molina and colleagues were the first studying *M. vastus lateralis* biopsies from human HFpEF patients in comparison to healthy controls (183). Compared with age-matched healthy control subjects, mitochondrial content assessed by porin expression was 46% lower, citrate synthase activity was 29% lower, and Mfn2 (mitofusin 2) expression was 54% lower in patients with HFpEF. Expression of porin was significantly positively correlated with both peakVO2 and 6-min walk distance and the expression of Mfn2 was also significantly positively correlated with both peakVO2 and 6-min walk distance.

Taken together it seems that also in HFpEF mitochondrial oxidative phosphorylation is impaired in the locomotor skeletal muscle, contributing to exercise intolerance. It also became evident that impairments in mitochondrial quality are more pronounced than the decrease in quantity.

Data on mitochondrial function and structure in the HFpEF diaphragm are limited to animal data. In the DSS rat model, *in situ* mitochondrial respiration of permeabilized diaphragm muscle fibers revealed that maximal ADP-stimulated respiration was reduced by 24% in HFpEF rats compared with controls (*p* < 0.05), indicating complex I dysfunction. In addition, the respiratory control ratio was impaired by 14% in HFpEF rats compared to controls (*p* < 0.05). However, enzyme activity of citrate synthase (reflecting mitochondrial volume density) was not different between controls and HFpEF (114). In the obese ZSF-1 rat model, saponin skinned fiber analysis revealed significant qualitative mitochondrial impairments, with HFpEF specifically increasing the leak state by ≈30% (*p* < 0.05) and reducing the respiratory control ratio by 20% (*p* < 0.05)—indicative of an impaired mitochondrial coupling efficiency (118).

In conclusion, mitochondrial dysfunction and structural abnormalities play a central role in the myopathy of the diaphragm in both HFrEF and HFpEF. In particular, human data are still scarce and further research is necessary to examine mitochondrial quantity and quality.

#### Inflammation and oxidative stress

Inflammation is a hallmark of HFrEF, and it is thought that the inflammatory status contributes to damage and dysfunction of the cardiovascular system, including the skeletal muscle, and even predicts clinical outcome (185, 186). With respect to origin of the elevated inflammatory cytokines, several hypotheses are discussed including production and secretion by circulating mononuclear cells, secretion by injured myocytes (187), or increased edema of the bowel wall and thereby induction of inflammation by lipopolysaccharide (188). During the development of HFrEF, an increase in circulatory inflammatory cytokines, especially tumor necrosis factor alpha (TNF-α), has been reported in either humans (189, 190) or animal models of HFrEF (191).

#### HFrEF

The question arises if this increase in inflammatory cytokines also contributes to the alterations observed in locomotor and respiratory muscle of HFrEF. Numerous studies clearly documented the catabolic effects of cytokines by activating the UPS (192–194) and promote contractile dysfunction in both the locomotor and diaphragm muscle by increasing ROS production (73, 195). Noteworthy, ROS induced diaphragmatic dysfunction may occur as early as three days after an acute myocardial infarction (196). Increased inflammatory cytokines are not only detected in the systemic circulation of HFrEF patients and HFrEF animal models, but the local mRNA expression of TNF-α, IL-6 and IL-1-beta in skeletal muscle myocytes is also increased and can be reduced by exercise training in HFrEF patients (72). Despite these overwhelming data on the pathophysiological association between inflammatory cytokines and HFrEF mortality and morbidity, the clinical trials targeting the causative role of inflammation in disease progression have been negative (reviewed in (197).

#### HFpEF

Importantly, the elevation of inflammatory cytokines is not only restricted to HFrEF but is also evident in HFpEF (97, 198). Even in hypertension, a diaphragm contractile dysfunction via an oxidant-mediated mechanism (mainly via NADPH oxidase) is evident and can be prevented by the anti-oxidative effects of exercise training (199). Inflammation and oxidative stress drives cardiomyocytes, endothelial cells, and skeletal muscle dysfunction. All of which are major determinants of diastolic dysfunction in HFpEF patients and animal models of HFpEF (200).

## Interventions for skeletal and respiratory muscle dysfunction in heart failure

Different pharmacological, exercise-based, and device-supported therapeutic options have been evaluated or are under investigation for skeletal muscle dysfunction in both HFrEF and HFpEF.

### Medical therapy

Low endogenous testosterone may represent an independent risk factor for HF (201). Treatment with testosterone as a potential strategy to counteract exercise intolerance and dyspnea in chronic HF has been investigated, describing positive results regarding improved exercise capacity (202) and a reduction of symptoms in HF patients (203). However, the safety of testosterone supplementation and its potential negative effects on the cardiovascular system have to be taken in consideration and further studies are necessary to evaluate the net benefit (204). Therefore, different selective androgen receptor modulators (SARMs) are currently being explored due to their potential anabolic activity but without side effects of androgens (204). However, data from large-scale studies confirming the potential muscle-protective effects of SARMs in HF patients are not available yet.

With regard to medical treatment of HF associated muscle dysfunction, supplementation of essential amino acids (8 g/day) have shown positive results regarding the physical performance, but did not increase absolute muscle mass in patients with stable chronic HF and severe loss of muscle mass (205). Some standard HF medications have demonstrated potential benefits against muscle loss. Angiotensin II-converting enzyme inhibitors (ACE-Is), due to their anti-oxidative and anti-inflammatory effects, could have muscle protective effects (206). In a sub-analysis of the Studies of Left Ventricular Dysfunction (SOLVD) (207), including 1,929 chronic HF patients, showed that patients taking enalapril had a 19% lower risk of developing cachexia. Beneficial effects of mineralocorticoid antagonists on skeletal muscle homeostasis have been reported (208) and some studies also suggest that beta-blockers may also slow down wasting processes associated with increased sympathetic activation (209). SGLT-2-inhibitors provide a true success story with a reduction of the composite endpoint of cardiovascular death and hospitalization for HF and its components in both HFrEF and HFpEF (210). With regard to the effect on muscle mass and function, data are still scarce. In HFpEF, treatment with empagliflozin significantly improved E/é and resulted in improved locomotor muscle contractility with reduced intramuscular lipid content as well as an improved mitochondrial function with only minor modulation of atrophy-related proteins (110). Analyzing the impact of Empagliflozin on muscle endurance in an LAD-ligation model of HFrEF, Nambu and colleagues documented a significant increase in muscle performance without any changes in muscle weight, probably related to a restoration of mitochondrial fatty acid oxidation (211).

Currently, compounds for wasting disorders in chronic HF are being tested in preclinical and clinical settings. Acylated ghrelin has a potential anti-catabolic effect, as demonstrated by an experimental study conducted in a chronic HFrEF rat model (175), possibly by regulation of the UPS rate-limiting E-3 ubiquitin ligases, muscle RING-finger protein-1 (MuRF-1) and Muscle Atrophy F-box (MAFbx)/atrogin-1 (212). Moreover, its intravenous administration in a small cohort of HFrEF patients underlined an amelioration of exercise capacity and muscle strength (213). Recently, treatment with the MuRF-1 inhibitor compound ID#704946 prevented diaphragm fiber atrophy as well as impaired contractile function, and reduced mitochondrial enzyme activities caused by both monocrotaline induced cardiac cachexia and post-MI HFrEF (101, 107). Moreover, the slightly modified und further developed compound MyoMed-205 improved myocardial diastolic function and prevented locomotor muscle atrophy/function in the ZSF1 animal model of HFpEF. Mechanistically, locomotor muscles benefited from an attenuated ubiquitin proteasome system and augmented synthesis/activity of proteins of the mitochondrial respiratory chain while the myocardium seemed to benefit from reduced titin modifications and fibrosis (101, 184).

### Exercise training

**Figure 3:**
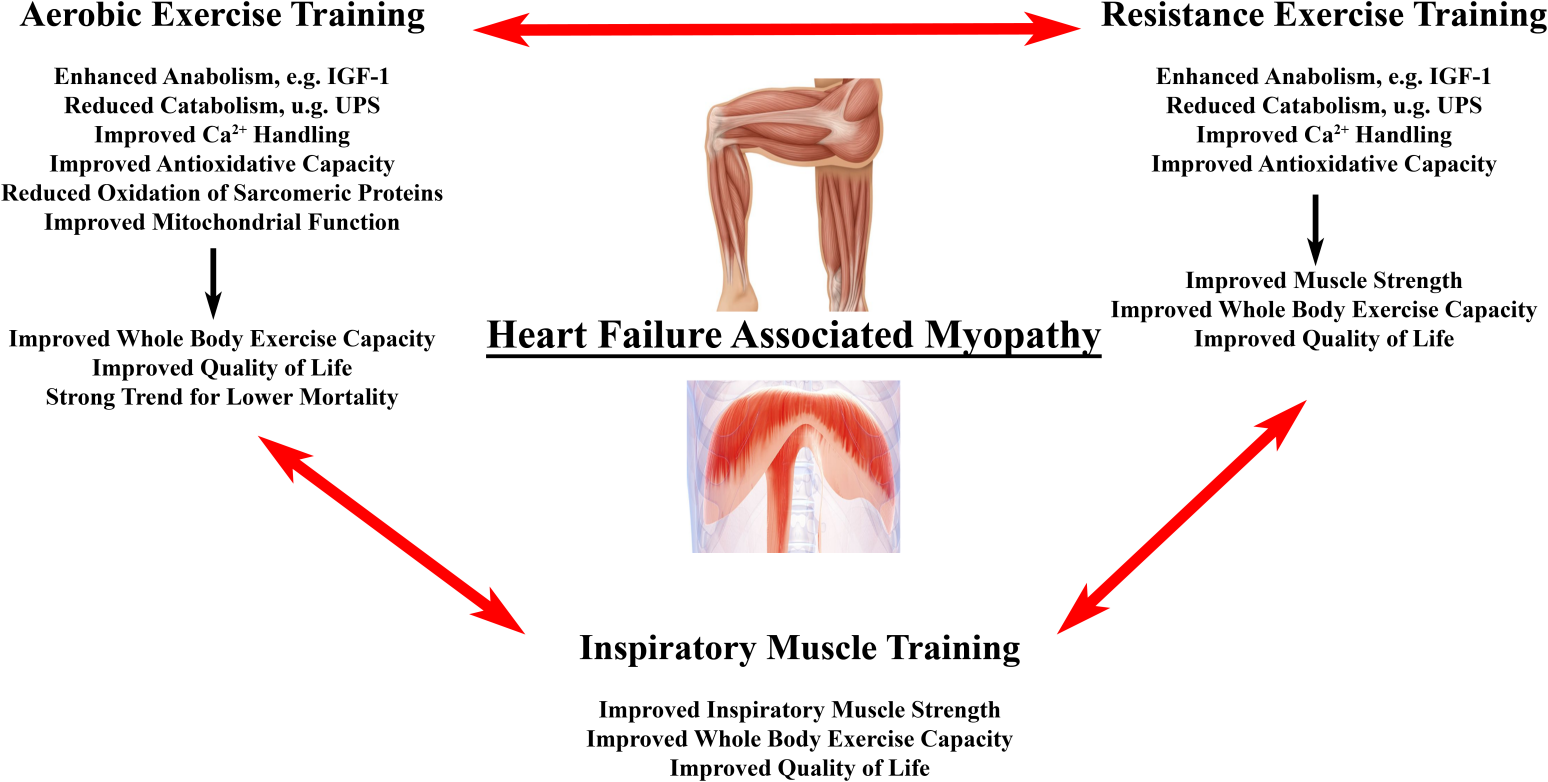
Effects of exercise training on heart failure associated myopathy. Primary effects of different forms of exercise training on locomotor and/or respiratory muscle changes in heart failure associated myopathy. Evidence suggests that a combination of all three modalities provides superior outcome with regard to surrogate endpoints in HF patients with the need for larger studies to confirm this finding (214).

Exercise training might be the “magic bullet” for HF associated muscle dysfunction including aerobic (AET) and high intensity interval training (HITT), resistance training (RET) and inspiratory muscle training (IMT) (Figure 3). Exercise training has been demonstrated to be a positive stimulus on muscle mass, muscle quality, and physical performance in patients with HF [for detailed review see (215, 216)]. In particular, AET is associated with improved quality of life, reduced HF hospitalizations, and prolonged survival (217). We will briefly review, how AET, RET and IMT may benefit HF related myopathy by impacting antioxidative measures, muscle mass, and contractile dysfunction.

In HF patients and animal models, AET led to anti-inflammatory effects and improved antioxidant enzyme expression, mainly by reducing the pro-inflammatory cytokines of tumor necrosis factor-α and interleukin-6 muscle expression (72, 218) and by increasing glutathione peroxidase 1 and catalase enzyme activities (218).

Moreover, AET is able to restore the protein synthesis-degradation imbalance by activating the IGF-1/Akt/mTORC1 pathway in both animals and humans with HF (31, 102), and, on the other hand, by reducing protein degradation via the UPS (31, 219, 220).

Positive effects of AET have been shown for HF-induced Ca^2+^ dysfunction of myofibers as indicated by an increased expression of RYR1 and SERCA proteins (221), with experimental models suggesting a link between improved exercise tolerance in HF with AET and restored expression of Ca^2+^-related proteins (222). Moreover, AET prevents oxidative contractile protein modifications of actin and creatine kinase in the diaphragm of HF mice, probably via increased activity and expression of antioxidant enzymes (119). Improvement of contractile dysfunction by exercise training is supported by a study describing increased muscle strength after RET in HF patients despite a lack of myofiber hypertrophy (223). In contrast, 4 weeks of RET in LAD-ligated mice restored locomotor muscle weight relative to body mass and muscle fiber area compared to controls and was associated with a reduction of E3-ligase expression to control levels, a decreased myostatin protein expression, and improved anabolic profile (224). Therefore, and despite initial safety concerns, RET has proven to be safe and improves parameters of physical function and quality of life. Therefore, RET should be substituted to AET or even considered as first choice in patients unable to perform AET (225).

Finally, there is evidence suggesting that IMT in HF patients is able to restore diaphragmatic and respiratory muscle function (226). In a meta-analysis, isolated IMT led to a statistically significant increase in Pi_max_ by 25 cm H_2_O as well as improved exercise capacity and quality of life (227).

However, the question about the best mode of exercise training in HF remains with exercise programs that consisted of combined AET, RET, and IMT showing superior outcomes to either AET/RET, AET/IMT, or AET alone in improving aerobic capacity and circulatory power in HFrEF patients (214). The effects on the cellular and molecular levels of the diaphragm in humans has not been studied; however, as mentioned above, AET averted contractile dysfunction of diaphragm fiber bundles and reduced markers of proteolysis and oxidative stress in a LAD-ligated mice (119).

### Device therapy

Finally, and in particular for diaphragm dysfunction, a device-based therapy has been established in the last years with the need for larger clinical trials to understand the clinical benefit of this therapy. For review please refer to Salah H.M. and colleagues (56) since the detailed description of this therapeutic option is beyond the scope of this review.

## Conclusion and future directions

In this review, we have demonstrated that HF independent of LV-EF leads to a secondary myopathy of both the locomotor and respiratory muscles contributing to exercise intolerance and reduced quality of life. Intensive research in the last decades revealed a better understanding of the underlying molecular mechanisms, which are the key for the development of therapeutic options for an increasing patient population due to an aging society with a high prevalence of HF. The mechanisms involved include HF-associated changes regarding muscle mass, contractile dysfunction, fiber type composition and capillarization, mitochondria as well as inflammation and reactive oxygen species. However, exercise training attenuates a number of these impairments, but beside the remaining question which exercise modality is most effective, it is an eminent task to implement permanent exercise programs in HF patients with a high rate of compliance. Different established and new medical therapies have been tested to treat specific changes in HF-associated myopathy with the aim to improve exercise capacity and quality of life. So far, the effects of pharmacological interventions are modest, and further research is necessary to elucidate treatment options in patients unable to perform physical activity.

## Author contributions

NM, EW, and VA wrote the first draft of the manuscript. NM, EW, VA, and AL provided critical feedback. All authors contributed to the article and approved the submitted version.
